# Clinical outcomes in patients co-infected with COVID-19 and *Staphylococcus aureus*: a scoping review

**DOI:** 10.1186/s12879-021-06616-4

**Published:** 2021-09-21

**Authors:** Jenna R. Adalbert, Karan Varshney, Rachel Tobin, Rafael Pajaro

**Affiliations:** 1 Sidney Kimmel Medical College at Thomas Jefferson University, Philadelphia, PA USA; 2 Jefferson College of Population Health, 901 Walnut St., Philadelphia, PA 19107 USA; 3 Deakin University School of Medicine, Geelong, Victoria Australia; 4 Morristown Medical Center of Atlantic Health System, Morristown, New Jersey USA

**Keywords:** COVID-19, *Staphylococcus aureus*, Co-infection, Antibiotics, Hospitalization, Infection

## Abstract

**Background:**

Endemic to the hospital environment, Staphylococcus aureus (*S. aureus*) is a leading bacterial pathogen that causes deadly infections such as bacteremia and endocarditis. In past viral pandemics, it has been the principal cause of secondary bacterial infections, significantly increasing patient mortality rates. Our world now combats the rapid spread of COVID-19, leading to a pandemic with a death toll greatly surpassing those of many past pandemics. However, the impact of co-infection with *S. aureus* remains unclear. Therefore, we aimed to perform a high-quality scoping review of the literature to synthesize the existing evidence on the clinical outcomes of COVID-19 and *S. aureus* co-infection.

**Methods:**

A scoping review of the literature was conducted in PubMed, Scopus, Ovid MEDLINE, CINAHL, ScienceDirect, medRxiv, and the WHO COVID-19 database using a combination of terms. Articles that were in English, included patients infected with both COVID-19 and *S. aureus*, and provided a description of clinical outcomes for patients were eligible. From these articles, the following data were extracted: type of staphylococcal species, onset of co-infection, patient sex, age, symptoms, hospital interventions, and clinical outcomes. Quality assessments of final studies were also conducted using the Joanna Briggs Institute’s critical appraisal tools.

**Results:**

Searches generated a total of 1922 publications, and 28 articles were eligible for the final analysis. Of the 115 co-infected patients, there were a total of 71 deaths (61.7%) and 41 discharges (35.7%), with 62 patients (53.9%) requiring ICU admission. Patients were infected with methicillin-sensitive and methicillin-resistant strains of *S. aureus*, with the majority (76.5%) acquiring co-infection with *S. aureus* following hospital admission for COVID-19. Aside from antibiotics, the most commonly reported hospital interventions were intubation with mechanical ventilation (74.8 %), central venous catheter (19.1 %), and corticosteroids (13.0 %).

**Conclusions:**

Given the mortality rates reported thus far for patients co-infected with *S. aureus* and COVID-19, COVID-19 vaccination and outpatient treatment may be key initiatives for reducing hospital admission and *S. aureus* co-infection risk. Physician vigilance is recommended during COVID-19 interventions that may increase the risk of bacterial co-infection with pathogens, such as *S. aureus*, as the medical community’s understanding of these infection processes continues to evolve.

**Supplementary Information:**

The online version contains supplementary material available at 10.1186/s12879-021-06616-4.

## Background

Upon passage of the March 11th anniversary of the official declaration of the coronavirus disease 2019 (COVID-19) pandemic [1], the causative severe acute respiratory syndrome coronavirus 2 (SARS-CoV-2) pathogen has infected over 181 million individuals and resulted in more than 3.9 million deaths worldwide as of July 1, 2021 [2]. In addition to rapid spread through high transmission rates [3], infection with COVID-19 can result in severe complications such as acute respiratory distress syndrome (ARDS), thromboembolic events, septic shock, and multi-organ failure [4]. In response to this novel virus, the clinical environment has evolved to accommodate the complexities of healthcare delivery in the pandemic environment [5]. Accordingly, a particularly challenging scenario for clinicians is the management of patients with common infections that may be complicated by subsequent COVID-19 co-infection, or conversely co-infected with a pathogen following primary infection with COVID-19 [6]. Bacterial co-infection in COVID-19 patients may exacerbate the immunocompromised state caused by COVID-19, further worsening clinical prognosis [7].

Implicated as a leading bacterial pathogen in both community- and healthcare-associated infections, *Staphylococcus aureus (S. aureus)* is commonly feared in the hospital environment for its risk of deadly outcomes such as endocarditis, bacteremia, sepsis, and death [8]. In past viral pandemics, *S. aureus* has been the principal cause of secondary bacterial infections, significantly increasing patient mortality rates [9]. For viral influenza infection specifically, *S. aureus* co-infection and bacteremia has been associated with mortality rates of almost 50%, in contrast to the 1.4% morality rates observed in patients infected with influenza alone [10]. Given the parallels between the clinical presentation, course, and outcomes of influenza and COVID-19 viral infection [11], mortality rates in COVID-19 patients co-infected with *S. aureus* may reflect those observed in influenza patients. However, while recent studies have focused on the incidence and prevalence of COVID-19 and *S. aureus* co-infection, the clinical outcomes of patients co-infected with these two specific pathogens remains unclear given that existing studies consolidate *S. aureus* patient outcomes with other bacterial pathogens [12–14].

Given that the literature informing our knowledge of COVID-19 is a dynamic and evolving entity, the purpose of this scoping review is to evaluate the current body of evidence reporting the clinical outcomes of patients co-infected with COVID-19 and *S. aureus*. To date, there has been no review focusing specifically on the clinical treatment courses and subsequent outcomes of COVID-19 and *S. aureus* co-infection. In response to the urgency of the pandemic state and high rates of COVID-19 hospital admissions, we aim to identify important areas for further research and explore potential implications for clinical practice.

## Methods

### Search strategy and study selection

To provide a scoping review of initial insight into the breadth of developing data on COVID-19 and *S. aureus* co-infection, we followed the five-stage methodology of scoping review practice presented by Levac, Colquhoun, and O’Brien [15]. In accordance with the Preferred Reporting Items for Systematic Reviews and Meta-Analyses (PRISMA) extension for Scoping Reviews [16], we conducted electronic searches in PubMed, Scopus, Ovid MEDLINE, CINAHL, ScienceDirect, medRxiv (preprint), and the WHO COVID-19 database between July 3, 2021 and July 16, 2021. Search terms were combined with the use of Boolean operators and included subject headings or key terms specific to COVID-19 (i.e. severe acute respiratory syndrome coronavirus 2 OR SARS-CoV2 OR 2019 novel coronavirus OR 2019-nCoV OR coronavirus disease 2019 virus OR COVID-19 OR Wuhan coronavirus) and *Staphylococcus aureus* (i.e. methicillin-resistant staphylococcus aureus OR MRSA OR methicillin-susceptible *Staphylococcus aureus* OR MSSA OR staphylococcal infections). A comprehensive list of our scoping terms and search strategies is included in the Appendix (Ädditional file 1: Table S1). Two independent, experienced reviewers (JA and KV) screened the titles and abstracts of eligible studies and performed full-text review on qualified selections. For this review, we broadly considered articles of any design that included patients infected with both COVID-19 and *S. aureus*, provided a description of the timeline and ultimate clinical outcomes for these patients (i.e. death or discharge from hospital) at study completion, and were available in English. Studies were excluded if they did not report final outcomes since our scoping review purpose was to evaluate the quality of existing literature that described the clinical course and mortality rate of patients co-infected with these pathogens. We excluded duplicate records and disagreements regarding study inclusion were resolved by consensus or feedback from the senior author.

### Data extraction

For the final articles selected, we completed data extraction in duplicate, and any discrepancies were resolved through discussion or consult with the senior author. While several studies also included reports on patients infected with COVID-19 alone or co-infected with an alternative pathogen, we extracted data solely for patients with COVID-19 and *S. aureus* co-infection. Our data extraction items included study methodology, author and study location, type of staphylococcal species, onset of *S. aureus* infection, *S. aureus* culture site and infection source, patient sample size, age, gender, presentation, comorbidities or additional co-infections, prior history of *S. aureus* infection, diagnostic findings, hospital treatments and interventions, complications, total length of hospital admission, intensive care unit transfer, and final patient mortality outcomes upon study completion.

### Data synthesis and analysis

Microsoft Excel 2016 (Redmond, WA, USA) was used to collect and chart data extracted from the studies that met the inclusion criteria. Data was synthesized and analyzed descriptively, with frequency counts performed for individual and grouped study metrics. The purpose of synthesizing the extracted information through this method was to create an overview of existing knowledge and identify gaps in the current literature on COVID-19 and *S. aureus* co-infection.

### Quality assessment

Given that the majority of existing literature reporting outcomes data for COVID-19 and *S. aureus* co-infection were case reports, we utilized the Joanna Briggs Institute’s critical appraisal tools [17] to provide a metric for our scoping assessment of the methodological quality of the included studies. Application of these tools enabled examination of study quality in the areas of inclusion criteria, sample size, description of study participants, setting, and the appropriateness of the statistical analysis. As in previous reviews [18, 19], the tools were modified to produce a numeric score with case reports assessed based on an eight-item scale, case series on a ten-item scale, and cohort studies on an eleven-item scale. Studies were assessed with the methodological quality tool specific to their design (i.e. case report, case series, cohort) by two independent reviewers (JA and KV) and discrepancies were resolved through discussion. While debate exists regarding the minimal number of patients required for study qualification as a “case series” [20], we considered studies reporting individual patient data as “case reports” and those reporting aggregate patient data as “case series.” Our complete quality assessment, including tools and scores, is available in the Appendix (Additional file 1: Tables S2–S4).

## Results

**Fig. 1:**
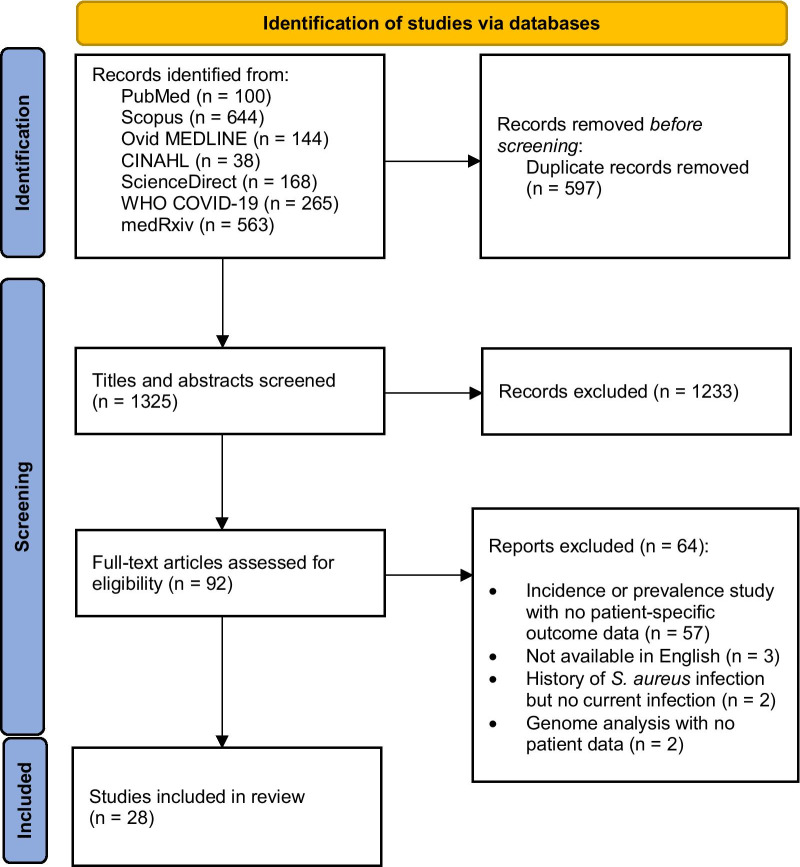
Process of searching and selecting articles included in the scoping review based on the PRISMA 2020 flow diagram

Our search strategy produced a total of 1922 potential publications with patients co-infected by COVID-19 and *S. aureus*. For transparent and reproducible methods, the PRISMA 2020 flow diagram for new systematic reviews was utilized to display the search results of our scoping review (Fig. 1). Following deduplication (n = 597) and a comprehensive screen of study titles and abstracts for irrelevant material (n = 1233), we reviewed 92 full texts for inclusion eligibility. Of these texts, 64 did not include patient outcomes for COVID-19 and *S. aureus* co-infected patients: 57 were incidence or prevalence studies with no patient-specific outcomes data, two included patients with COVID-19 and a history of *S. aureus* infection but no current COVID-19 and *S. aureus* co-infection, two were genome analysis studies with no patient data, and three were unavailable in English (Additional file 1: Table S5).

### Publication types and geography

Following full-text review, 28 studies qualified for inclusion in our review, resulting in a total of 115 patients. Of these 28 included studies, 22 were case reports (describing single patients with individual data), two were case series (describing 7–42 patients with aggregate data), and four were cohort studies (describing 4–40 patients with aggregate data). Countries of study publication included the United States (n = 7) [7, 9, 21–25], Italy (n = 7) [26–32], Japan (n = 2) [33, 34], Iran (n = 2) [35, 36], the United Kingdom (n = 2) [37, 38], Spain (n = 2) [39, 40], Bahrain (n = 1) [41], China (n = 1) [42], France (n = 1) [43], the Philippines (n = 1) [44], Korea (n = 1) [45], and Canada (n = 1) [46], with publication dates ranging from April 15, 2020 to June 16, 2021. Table 1 describes the characteristics of these included studies and available information on their respective patient demographics in detail.

**Table 1:** Study and patient characteristics

First Author	Country	Publication date	Study design	Quality Assessment	N	Age	Male/Female	Type	Co-infection	Comorbidities	Outcome
Adachi	Japan	05/15/20	Case report	8/8	1	84	Female	MSSA	*Klebsiella pneumoniae*	None	Death
Bagnato	Italy	08/05/20	Case report	8/8	1	62	Female	MSSA	*Candida tropicalis*	Hypertension	Discharge
Chandran	United Kingdom	05/20/21	Case report	7/8	1	NR	Female	MSSA	None	Type 2 diabetes mellitus	Death
Chen	China	11/01/20	Case report	6/8	1	29	Male	MSSA and MRSA	*Haemophilus influenzae*	NR (not reported)	Discharge
Choudhury	USA	07/04/20	Case report	7/8	1	73	Male	MSSA	None	Type 2 diabetes mellitus, chronic foot osteomyelitis, aortic stenosis, prosthetic aortic valve, atrial fibrillation, prior *S. aureus* infection	Hospice
Cusumano	USA	11/12/20	Case series	9/10	42	65.6 (mean)	Males (n = 21), Females (n = 21)	MSSA (n = 23) and MRSA (n = 19)	*Enterococcus faecalis* (n = 3), *Candida* spp. (n = 2), *Klebsiella pneumoniae* (n = 2), *Escheria coli* (n = 1), *Bacillus* spp. (n = 1), *Micrococcus* spp. (n = 1), *Staphylococcus epidermidis* (n = 1), *Proteus mirabilis* (n = 1)	Hypertension (n = 29), diabetes mellitus (n = 21), cardiovascular disease (n = 19), lung disease (n = 7), chronic kidney disease (n = 6), malignancy (n = 5), end-stage renal disease (n = 4), organ transplant (n = 3), liver disease (n = 1)	Death at 30 days (n = 28)
De Pascale	Italy	05/31/21	Prospective cohort	8/11	40	64 (mean)	Males (n = 33), Females (n = 7)	MSSA (n = 14), MRSA (n = 26)	Bacteroidetes (n = 18), Proteobacteria (n = 7), Actinobacteria (n = 3), Tenericutes (n = 2), Fusobacteria (n = 1)^1^	Diabetes mellitus (n = 8), cardiovascular disease (n = 7), lung disease (n = 7), immunosuppression (n = 4), neoplasm (n = 4), chronic kidney disease (n = 3)	Death (n = 26)
Duployez	France	04/16/20	Case report	8/8	1	35	Male	MSSA (PVL-secreting)	None	None	Death
Edrada	Philippines	05/07/20	Case report	6/8	1	39	Female	MSSA	*Influenza B*, *Klebsiella pneumoniae*	None	Discharge
ElSeirafi	Bahrain	06/23/20	Case report	6/8	1	59	Male	MRSA	None	NR	Death
Filocamo	Italy	05/11/20	Case report	8/8	1	50	Male	MSSA	None	None	Discharge
Hamzavi	Iran	08/01/20	Case report	6/8	1	14	Male	MSSA	None	Cerebral palsy	Death
Hoshiyama	Japan	11/02/20	Case series	6/10	1	47	Male	MSSA	None	Previous cerebral hemorrhage	Discharge
“”	“”	“”	“”	“”	1	39	Male	MSSA	*Group B Streptococcus*	Hypertension	Discharge
Hussain	United Kingdom	05/22/20	Case report	8/8	1	69	Female	MSSA	None	Prosthetic aortic valve with reduced ejection fraction	Death
Levesque	Canada	07/01/20	Case report	6/8	1	53	Female	MSSA	None	Hypertension, diabetes mellitus, dyslipidemia	Hospital
Mirza	USA	11/16/20	Case report	6/8	1	29	Male	MRSA	Multi-drug resistant *Pseudomonas*	Cystic fibrosis with moderate obstructive lung disease, exocrine pancreatic insufficiency, gastroparesis, chronic *S. aureus*	Discharge
Patek	USA	04/15/20	Case report	7/8	1	0	Male	MSSA	Herpes simplex virus	Maternal history of oral herpetic lesions	Discharge
Posteraro	Italy	09/06/20	Case report	8/8	1	79	Male	MRSA	*Morganella morganii*, *Candida glabrata*, *Acinetobacter baumannii*, *Proteus mirabilis*, *Klebsiella pneumoniae, Escherichia coli*	Type 2 diabetes mellitus, ischemic heart disease, peripheral artery disease, left leg amputation	Death
Rajdev	USA	09/10/20	Case report	7/8	1	32	Male	MSSA	*Klebsiella pneumoniae*	Type 2 diabetes mellitus	Discharge
Rajdev	USA	09/28/20	Case report	7/8	1	36	Male	MSSA	*Haemophilus influenzae*	Hypertension, two renal transplants for renal dysplasia	Discharge
Ramos-Martinez	Spain	07/30/20	Prospective cohort	6/11	1	60	NR	MSSA	None	Type 2 diabetes mellitus, hypercholesterolemia, wrist arthritis, sternoclavicular arthritis	Death
Randall	USA	12/01/20	Case report	7/8	1	60	Male	MRSA	None	Chronic obstructive lung disease, coronary artery disease, hypothyroidism	Death
“”	“”	“”	“”	“”	1	83	Male	MRSA	None	Hypertension, atrial fibrillation	Death
“”	“”	“”	“”	“”	1	60	Male	MRSA	Hepatitis C	Hypertension, type 2 diabetes mellitus, cirrhosis	Death
Regazzoni	Italy	08/07/20	Case report	2/8	1	70	Male	MSSA	None	NR	Hospital
Sharifipour	Iran	09/01/20	Prospective cohort	7/11	1	NR	NR	MSSA	None	None	Discharge
	“”	“”	“”	“”	1	NR	NR	MRSA	None	Type 2 diabetes mellitus	Death
Son	Korea	06/16/21	Retrospective cohort	8/11	4	79 (mean)	Male (n = 3), Female (n = 1)	MRSA	*C. albicans* (n = 2), Vancomycin-resistant enterococci (n = 2), *S. maltophilia* (n = 1) carbapenem-resistant *Acinetobacter baumannii* (n = 1),	NR	Death (n = 3)
Spannella	Italy	06/23/20	Case report	8/8	1	95	Female	MSSA	*Citrobacter werkmanii*	Hypertension, chronic heart failure, paroxysmal atrial fibrillation, dyslipidemia, chronic kidney disease, vascular dementia, sacral pressure ulcers, dysphagia	Death
Spoto	Italy	09/30/20	Case report	6/8	1	55	Female	MSSA	None	Triple negative, *BRCA1*-related, right breast cancer with multiple bone metastasis, type 2 diabetes mellitus	Death
Valga	Spain	06/11/20	Case report	6/8	1	68	Male	MSSA	None	Hypertension, type 2 diabetes mellitus, congestive heart failure, sleep apnea, ischemic heart disease, chronic kidney disease	Discharge

Patients were colonized with these bacterial phyla, but no distinction between colonization versus infection was reported

### Publication quality

**Fig. 2:**
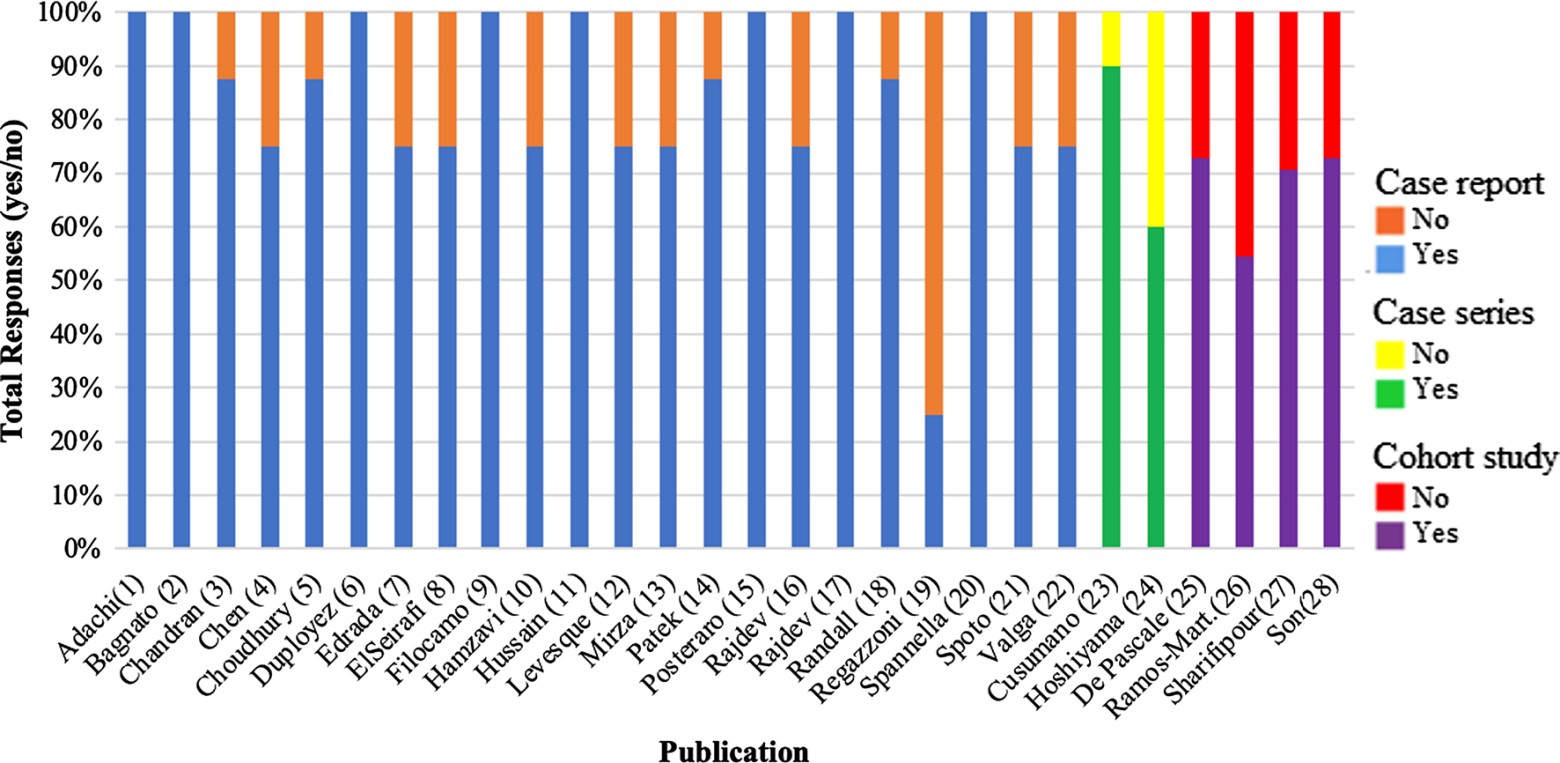
Quality assessment scores for included publications reported as “yes” or “no” for achieving quality metrics per the Joanna Briggs Institute’s critical appraisal tools

Figure 2 represents the quality assessment scores produced by the Joanna Briggs Institute’s critical appraisal tools. Scores ranged from 2 to 8 for case reports (out of 8 points total) (n = 22), 6–9 for case series (out of 10 points total) (n = 2), and 6–8 for cohort studies (out of 11 points total) (n = 4). The mean quality assessment score for these publications compared within their respective categories was 6.8 for case reports, 7.5 for case series, and 7.3 for cohort studies. In terms of most common study design limitations, the metric of patient post-intervention clinical conditions was least clearly described for case reports, neither of the case series consecutively included participants, and strategies to address incomplete follow-up were only reported for one of the four cohort studies.

### Patient demographics

For the 115 total patients included in our review that were co-infected with COVID-19 and *S. aureus*, their demographic (Table 1) and clinical data (Table 2) were described with varying completeness. Staphylococcal species and patient outcomes are reported in both tables to enable direct comparison with patient demographics and clinical course. Across our patient sample, the mean patient age was 54.8 years (SD = 21.6), 65.3% (n = 75) were male, 32.1% (n = 37) were female, and 3 patients (2.6%) did not have their gender specified in the study. Patients presented with a diversity of comorbidities with diabetes mellitus (33.9%, n = 39), hypertension (32.2%, n = 37), and cardiovascular disease (28.7%, n = 33) reported as the most common. Five patients presented with no comorbidities and four studies reported no information on patient medical history related to comorbidities. The most common presenting symptoms reported by patients at hospital admission included cough (13.9%, n = 16), fever (13.9%, n = 16), and dyspnea (13.0%, n = 15).

**Table 2:** Clinical characteristics

First Author	N	Type	Diagnosis	Co-infection onset	Presentation	Dx findings	Treatments and Interventions	Complications	Length of stay	ICU	Outcome
Adachi	1	MSSA	Sputum sample, pneumonia	Unclear^a^	Fever, diarrhea, dyspnea	Bilateral opacities on chest x-ray (CXR), ground glass opacities & lower lobe consolidation on chest computed tomography (CT)	Antibiotics, corticosteroids, lopinavir/ritonavir, morphine	ARDS	16	Yes	Death
Bagnato	1	MSSA	Blood culture, bacteremia	Hospital-onset	Fever, cough, diarrhea, myalgia	Unremarkable head CT, normal creatine kinase	Antibiotics, corticosteroids, intubation and ventilation, antifungals, lopinavir/ritonavir, hydroxychloroquine, tocilizumab, neuromuscular blocking agents, olanzapine	Psychomotor agitation and temporospatial disorientation, myopathy	140	Yes	Discharge
Chandran	1	MSSA	Blood culture and tracheal aspirate, pneumonia (ventilator-associated) and bacteremia	Hospital-onset	Dyspnea (positive COVID test)	Bilateral interstitial infiltrates (CXR) and ground glass opacities (CT)	Antibiotics, intubation and ventilation	Bilateral cavitating lung lesions, septic shock	15	Yes	Death
Chen	1	MSSA and MRSA	Sputum sample, pneumonia	Hospital-onset	Asymptomatic (positive COVID test)	Patchy consolidation and ground glass opacities in right upper lobe on CXR (day 29)	Antibiotics, corticosteroids, lopinavir/ritonavir, Abidol combined with IFN inhalant, Thymalfasin, ribavirin, loratadine	Pneumonia	51	No	Discharge
Choudhury	1	MSSA	Blood culture, endocarditis and bacteremia	Unclear^b^	Altered mental status, low back pain, urinary incontinence, right foot ulcers	Cystitis and pyelonephritis on CT, epidural abscess (L4/5) on magnetic resonance imaging (MRI)	Antibiotics, oral rifampin, hydroxychloroquine	Endocarditis, aortic root abscess	NR (not reported)	NR	Hospice
Cusumano	42	MSSA (n = 23) and MRSA (n = 19)	Blood culture, bacteremia (n = 42), pneumonia (n = 8), vascular (n = 3), osteomyelitis (n = 1), skin (n = 1)	Hospital-onset (n = 28), community-onset (n = 14)	Not reported (NR)	Abnormal CXR (n = 36), vegetation on transthoracic echo (n = 1)	Antibiotics (n = 42), intubation and ventilation (n = 31), central venous catheter (n = 19)	NR	NR	NR	Death at 30 days (n = 28)
De Pascale	40	MSSA (n = 14), MRSA (n = 26)	Tracheal aspirate and blood culture, pneumonia (ventilator-associated) (n = 40) and bacteremia (n = 19)	Late hospital-onset (n = 35), early hospital-onset (n = 5)	NR	NR	Antibiotics (n = 40), intubation and ventilation (n = 40)	Septic shock (n = 22), acute kidney injury (n = 4)	11 (mean)	Yes (n = 40)	Death (n = 26)
Duployez	1	MSSA (PVL-secreting)	Pleural drainage sample, pneumonia	Unclear^c^	Fever, cough, bloody sputum	Consolidation of left upper lobe, left pleural effusion, right ground glass opacities, bilateral cavitary lesions on CT	Antibiotics, intubation and ventilation, extracorporeal membrane oxygenation (ECMO), anticoagulation, upper left lobectomy	Necrotizing pneumonia, deterioration of respiratory, renal, and liver functions	17	Yes	Death
Edrada	1	MSSA	Nasal and throat swab with PCR	Community-onset/carrier	Dry cough, sore throat	Unremarkable chest CT	Oseltamivir	None	19	No	Discharge
ElSeirafi	1	MRSA	Blood culture, bacteremia	Hospital-onset	Fever, dry cough, dyspnea	Bilateral pulmonary infiltrates and ARDS on CXR	Antibiotics, IFN, ribavirin, plasma therapy, tocilizumab injections	Septic shock with multi-organ dysfunction	16	Yes	Death
Filocamo	1	MSSA	Blood culture, bacteremia	Hospital-onset	Fever, dyspnea	Bilateral ground glass opacities on chest CT	Antibiotics, intubation and ventilation, lopinavir/ritonavir, hydroxychloroquine, anakinra	Progressive cholestatic liver injury	29	Yes	Discharge
Hamzavi	1	MSSA	Blood culture, bacteremia	Unclear^d^	Fever, cough, dyspnea, lethargy	Left pleural effusion on CXR	Antibiotics, intubation and ventilation	Multi-organ dysfunction	NR	Yes	Death
Hoshiyama	1	MSSA	Throat swab and sputum sample	Unclear^e^	Cough	Normal labs	NR	NR	NR	No	Discharge
“”	1	MSSA	Throat swab and sputum sample	Unclear^e^	Cough	Normal labs	NR	NR	NR	No	Discharge
Hussain	1	MSSA	Blood culture, bacteremia	Community-onset	Fever, cough, dyspnea, malaise	Bilateral reticular enhancement and heavily calcified aortic valve with mass effect on left atrial wall on chest CT	Antibiotics, intubation and ventilation, esophagogastroduodenoscopy, pantoprazole, amiodarone, heparin	Bleeding Dieulafoy’s lesion, fast atrial fibrillation, acute kidney injury, multi-organ failure, intracerebral hematoma	18	Yes	Death
Levesque	1	MSSA	Sputum sample, pneumonia (ventilator-associated)	Hospital-onset	Fever, dry cough, dyspnea	Small intraventricular hemorrhage on head CT (day 39)	Antibiotics, intubation and ventilation, corticosteroids, propofol, fentanyl, neuromuscular blocking agents, heparin, continuous platelet infusion, blood transfusions, IVIG, endobronchial clot removals, romiplostim, vincristine	ARDS, ICU-acquired neuromyopathy, acute kidney injury, thrombocytopenia, intraventricular hemorrhage, ventilator-associated pneumonia	At least 39	Yes	Hospital
Mirza	1	MRSA	Sputum sample	Carrier (chronic)	Chest pain, dyspnea	Bilateral upper lobe bronchial wall thickening and bronchiectasis with nodular and interstitial opacities on chest CT	Antibiotics, remdesivir	Meropenem-resistant *pseudomonas*	6	No	Discharge
Patek	1	MSSA	Wound culture, cellulitis	Community-onset	Fever, erythema and erosions of right thumb and fourth digit, somnolence, decreased feeding	Elevated LFTs, bilateral perihilar streaking on CXR, neutropenia	Antibiotics, acyclovir, nasal cannula O2	Hypoxic respiratory failure	7	Yes	Discharge
Posteraro	1	MRSA	Blood culture, bacteremia	Hospital-onset	Fever, cough, dyspnea	CXR and chest CT consistent with pneumonia	Antibiotics, antifungals, hydroxychloroquine, darunavir/ritonavir	Hypoxia, left leg re-amputation, septic shock	53	Yes	Death
Rajdev	1	MSSA	Sputum sample, pneumonia (ventilator-associated)	Community- and hospital-onset^f^	Dyspnea	Bilateral consolidations on CXR, bilateral ground glass opacities and pneumomediastinum with subcutaneous emphysema on chest CT	Intubation and ventilation, epoprostenol, hydromorphone, neuromuscular blocking agents, ECMO	Anemia, epistaxis, oropharyngeal bleeding, ARDS	47	Yes	Discharge
Rajdev	1	MSSA	Tracheal aspirate, pneumonia	Hospital-onset	Fever, cough, dyspnea, myalgias	Diffuse bilateral pulmonary opacities on CXR	Antibiotics, intubation and ventilation, corticosteroids, tacrolimus, mycophenolate, remdesivir	Hypoxic respiratory failure, Guillan Barré syndrome	23	NR	Discharge
Ramos-Martinez	1	MSSA	Blood culture, bacteremia (central venous catheter-associated)	Hospital-onset	Fever, meningitis, right infrapopliteal deep vein thrombosis	Mild mitral insufficiency on transthoracic echo	Antibiotics, intubation and ventilation, central venous catheter, corticosteroids, tocilizumab	Native valve endocarditis, progressive sepsis	At least 20	Yes	Death
Randall	1	MRSA	Blood culture, bacteremia	Hospital-onset	Fever, cough, dyspnea	NR	Intubation and ventilation, corticosteroids, central venous catheter	Respiratory distress	3	NR	Death
“”	1	MRSA	Blood culture, bacteremia	Hospital-onset	Hypoxia (positive COVID test)	NR	Corticosteroids, remdesivir, central venous catheter	Septic shock	14	NR	Death
“”	1	MRSA	Blood culture, bacteremia	Hospital-onset	Hypoxia (positive COVID test)	NR	Corticosteroids	Cardiac arrest	10	Yes	Death
Regazzoni	1	MSSA	Nasal swab and blood culture, bacteremia	Hospital-onset	Bilateral pneumonia (positive COVID test)	Ischemic areas with hemorrhagic transformation on head CT and MRI, large vegetations on aortic valve with regurgitation on transesophageal echo	Antibiotics, corticosteroids	Severe systemic inflammatory response	At least 10	NR	Hospital
Sharifipour	1	MSSA	Tracheal aspirate, pneumonia (ventilator-associated)	Hospital-onset	Cough, dyspnea, sore throat	NR	Antibiotics, intubation and ventilation	Ventilator-associated pneumonia	13	Yes	Discharge
“”	1	MRSA	Tracheal aspirate, pneumonia (ventilator-associated)	Hospital-onset	Cough, dyspnea, sore throat	NR	Antibiotics, intubation and ventilation	Ventilator-associated pneumonia	9	Yes	Death
Son	4	MRSA	Sputum sample, pneumonia (n = 4)	Hospital-onset (n = 4)	Pneumonia (positive COVID test)	NR	Antibiotics (n = 4), corticosteroids (n = 4)	NR	42 (mean)	Yes	Death (n = 3)
Spannella	1	MSSA	Bronchoalveolar lavage, pneumonia	Community-onset	Fever, cough, emesis	Bilateral ground glass opacities and multiple areas of consolidation on CXR	Antibiotics, metoprolol, amiodarone, continuous positive-pressure airway	Atrial fibrillation, respiratory failure, altered mental status, tachycardia, severe hypoxemia	27	Yes	Death
Spoto	1	MSSA	Tracheal aspirate, pneumonia	Unclear^g^	Fever, dyspnea, respiratory distress following chemoimmunotherapy	Bilateral ground glass opacities and consolidation in the middle/upper lobes on chest CT	Antibiotics, intubation and ventilation, lopinavir-ritonavir, hydroxychloroquine	ARDS	5	NR	Death
Valga	1	MSSA	Tracheal aspirate, pneumonia	Hospital-onset	Fever, dry cough	NR	Antibiotics, intubation and ventilation, corticosteroids, hydroxychloroquine, lopinavir/ritonavir, IFN beta, heparin	ARDS, multi-organ failure	47	Yes	Discharge

^a^Positive sputum culture on day 10

^b^Patient recently treated for *S. aureus* prior to admission, but setting is unclear

^c^Pleural fluid tested on day 4

^d^Timeline of blood culture unclear

^e^Timeline of sputum testing unclear

^f^Positive sputum on admission, subsequent ventilator-associated infection

^g^Patient was receiving routine treatments in a healthcare-setting

### Infection characteristics

In terms of specific staphylococcal species co-infection, 51.3% (n = 59) of patients were infected with methicillin-sensitive staphylococcus aureus (MSSA) and 49.6% (n = 57) were infected with methicillin-resistant staphylococcus aureus (MRSA), with a single patient co-infected with both MRSA and MSSA. One patient co-infected with MSSA had a fatal Panton-Valentine Leukocidin toxin-producing strain of MSSA (PVL-MSSA). In addition to COVID-19 and *S. aureus* co-infection, 26.1% (n = 30) of patients were co-infected with one or more separate pathogens such as *Klebsiella pneumoniae* (n = 6), *Candida* spp. (n = 6), *Enterococcus* spp. (n = 5), *Haemophilus influenzae* (n = 2), *Proteus mirabilis* (n = 2), *Escherichia coli* (n = 2). Comprehensive patient co-infection data are reported in Table 1.

### Diagnoses and treatments

Of all 115 reported cases of co-infection with COVID-19 and *S. aureus*, diagnosis of *S. aureus* infection was most frequently established by blood culture in our patient sample (64.3%, n = 74), with *S. aureus* infections manifesting predominantly in patients as bacteremia (64.3%, n = 74) and pneumonia (55.7%, n = 64), accompanied by several additional endocarditis/vasculitis (3.5%, n = 4), cellulitis (1.7%, n = 2), and osteomyelitis (0.9%, n = 1) cases. Additionally, two patients that tested positive for *S. aureus* with no clear infection source were suspected to be chronic carriers of the bacterial pathogen. From this variety of infection presentations, the majority (76.5%, n = 88) experienced hospital-onset *S. aureus* co-infection following hospitalization for an initial infection with COVID-19, and 19 patients (16.5%) presented with *S. aureus* infection at the time of admission that was determined to be community-onset in etiology. Aside from a standard course of antibiotics, patients received a diversity of adjuvant treatments during their hospital admission, with the most common interventions including intubation and mechanical ventilation (74.8%, n = 86), a central venous catheter (19.1%, n = 22), and corticosteroids (13.0%, n = 15). Table 2 describes the clinical course following hospital admission for each patient in comprehensive detail.

## Complications and outcomes

During the hospital course of the 115 co-infected patients in our review, the most common complications were sepsis or systemic inflammatory response syndrome (23.5 %, n = 27), acute kidney injury (5.2%, n = 6), acute respiratory distress syndrome (4.3%, n = 5), pneumonia (4.3%, n = 5), and multi-organ dysfunction or failure (4.3%, n = 5). Transfer to an intensive care unit during admission was clearly reported for 53.9% (n = 62) of patients, unnecessary for 4.3% (n = 5), and not reported for the remaining 41.8% (n = 48). Patients were admitted for a mean length of 26.2 days (SD = 26.7) to any type of inpatient hospital unit, with the length of hospital stay not reported in five cases. Upon analysis of the final outcomes reported for the hospital course of our co-infected COVID-19 and *S. aureus* patient sample, 71 (61.7%) patients died, 41 (35.7%) were discharged, two remained hospitalized and in stable condition on study conclusion, and one patient was placed in hospice care. Table 2 further details the specific complications presenting in each patient’s hospital trajectory and Table 3 reports the final pooled frequencies of patient co-infection characteristics and outcomes.

**Table 3:** Pooled frequencies of patient co-infection characteristics and outcomes (n = 115)

	Total (%)
Gender	
Male	75 (65.3 )
Female	37 (32.1)
Unspecified	3 (2.6)
*Staphylococcal Species*	
MSSA	59 (51.3)
MRSA	57 (49.6)
Co-infection	
*Klebsiella pneumoniae*	6 (5.2)
*Candida* spp.	6 (5.2)
*Enterococcus* spp.	5 (4.3)
*Hemophilus influenzae*	2 (1.7)
*Escherichia coli*	2 (1.7)
*Proteus mirabilis*	2 (1.7)
*Acinetobacter baumannii*	2 (1.7)
*Bacillus* spp.	1 (0.9)
*Staphylococcus epidermidis*	1 (0.9)
*Micrococcus* spp.	1 (0.9)
*Pseudomonas* spp.	1 (0.9)
*Morganella morganii*	1 (0.9)
*Citrobacter werkmanii*	1 (0.9)
*S. maltophilia*	1 (0.9)
Hepatitis C	1 (0.9)
Herpes simplex virus	1 (0.9)
Group B Streptococcus	1 (0.9)
None	83 (72.2)
* S. Aureus* Diagnostic Test	
Blood culture	74 (64.3)
Tracheal aspirate	46 (40.0)
Sputum sample	11 (9.5)
Nasal swab	2 (1.7)
Lower respiratory tract sample	2 (1.7)
Chronic carrier	2 (1.7)
Wound culture	1 (0.9)
*S. aureus* Diagnosis	
Bacteremia	74 (63.4)
Pneumonia	64 (55.7)
Ventilator-associated	44 (38.3)
Endocarditis/vasculitis	4 (3.5)
Cellulitis	2 (1.7)
Chronic carrier	2 (1.7)
Osteomyelitis	1 (0.9)
Not reported	2 (1.7)
*S. Aureus* Infection Onset	
Hospital	88 (76.5)
Community	19 (16.5)
Unclear	7 (6.1)
Complications	
Sepsis/Systemic Inflammatory Response Syndrome	27 (23.5)
Acute kidney injury	6 (5.2
Acute respiratory distress syndrome	5 (4.3)
Pneumonia	5 (4.3)
Multi-organ dysfunction/failure	5 (4.3)
Bleeding/coagulopathy	5 (4.3)
Hypoxic respiratory failure	3 (2.6)
Myopathy/neuropathy	3 (2.6)
Abscess formation	2 (1.7)
Confusion and altered mental status	2 (1.7)
Atrial fibrillation	2 (1.7)
Endocarditis	2 (1.7)
Anemia	1 (0.9)
Cardiac arrest	1 (0.9)
Thrombocytopenia	1 (0.9)
Re-amputation	1 (0.9)
Cholestatic liver injury	1 (0.9)
Not reported	3 (2.6)
ICU	
Yes	62 (53.9)
No	5 (4.3)
Not reported	48 (41.8)
Outcome	
Death	71 (61.7)
Discharge	41 (35.7)
Hospital	2 (1.7)
Hospice	1 (0.9)

## Discussion

As our evidence base of the outcomes of patients with COVID-19 infection continues to expand, thorough review of the various clinical scenarios and environments inherent to the treatment process of this disease are crucial for patient care management and improvement. Given that higher levels of morbidity and death have been observed in influenza patients co-infected with multiple pathogens during past pandemics [47], exploring the outcomes of co-infected COVID-19 patients may establish similar trends and reveal strategies for decreasing the morbidity and mortality of this population in our current pandemic. Our review of the available clinical data reporting the outcomes of patients co-infected with COVID-19 and the common bacterial pathogen, *S. aureus*, was purposed to augment this knowledge base and has produced several key findings regarding mortality rate, co-infection onset, and treatment considerations for these patients.

Foremost, the mortality rate in our review for patients co-infected with COVID-19 and *S. aureus* was 61.7%, which depicts a significantly increased mortality rate when contrasted with patients infected solely by COVID-19 [48]. This outcome is comparable to the increased morality rates observed in patients acquiring co-infection with *S. aureus* in addition to influenza [10], however, our findings emphasize an important difference in the etiology of COVID-19 and influenza co-infection with *S. aureus*. For influenza specifically, co-infection with *S. aureus* is predominantly diagnosed upon patient presentation to a healthcare setting, indicating that the community is a frequent and supportive environment for the co-infection processes of these pathogens [9, 49]. In contrast, our findings indicate that co-infection with *S. aureus* predominantly occurs in the hospital environment for patients with COVID-19 infection. The terminology used to differentiate these infection etiologies is “community-associated” versus “healthcare-associated,” with delineation between these diagnoses occurring at 48-hours after admission to a hospital or healthcare facility [50]. Given that co-infection with COVID-19 and *S. aureus* occurred after hospital admission in 76.5% of the patients in our review, preventative measures in the community-setting or treatment in an outpatient environment may be important considerations for mortality reduction from healthcare-associated *S. aureus* infection.

Importantly, while the predominance of *S. aureus* co-infections occurring after patient admission for COVID-19 infection is likely associated with a wide diversity of patient- and environment-specific factors, our findings suggest that this infection sequence may be partly attributed to the COVID-19 treatment course. The most common patient interventions identified in our review included intubation and mechanical ventilation, central venous catheter placement, and corticosteroids, which are each associated with increased risks of bacterial infection through introduction of a foreign body or immunosuppressive properties that dually support bacterial growth [51, 52]. Although these first-line treatments for decompensating patients that present with severe COVID-19 infection may predispose patients to *S. aureus* bacterial co-infection and subsequently increased mortality rates, they are often unavoidable during the patient treatment course. Vigilant management surrounding these interventions in patients with COVID-19 infection, such as timely central line or ventilator removal and prudent steroid dosing, are key quality improvement practices that warrant routine physician adherence during patient treatment processes given co-infection mortality rates.

In contrast to COVID-19 infection alone, the increased patient morbidity and mortality of COVID-19 and healthcare-associated *S. aureus* co-infection identified in our review have important implications for future research and clinical practice. While of clear and crucial public health importance, our findings further emphasize the imperative of COVID-19 vaccination to reduce both infection and symptom severity that may predispose patients to the necessity of hospital interventions and subsequent *S. aureus* co-infection. The effectiveness of this strategy is exemplified by the reduction in influenza and *S. aureus* pathology observed with increased influenza vaccination [53, 54]. As seen with influenza co-infection, vaccination may be a crucial harm reduction measure given that no *S. aureus* prophylaxis exists, and the incidence of *S. aureus* strains refractory to antibiotics is rising [55]. Additionally, the mortality trends observed in COVID-19 patients co-infected with *S. aureus* highlight the necessity for future reviews and clinical studies focused on the co-infection outcomes of other bacterial and viral pathogens alongside COVID-19. Further research may inform our ability to predict the trajectory of patients with various co-infections and identify infection patterns that influence treatment decisions.

To our knowledge, this is the first study to review and evaluate the outcomes of patients co-infected with COVID-19 and *S. aureus*. However, we acknowledge several limitations to this review. First, the majority of the studies included in our review were individual case reports due to the recent emergence of COVID-19 and limited literature exploring outcomes for patients co-infected with *S. aureus*. While these types of studies can be vital for expanding the medical knowledge base and reveal fundamental disease characteristics, it is crucial to consider the reporting bias that may exist in this study design and lack of comparison groups. Per our quality assessment, trends in study limitations for each type of publication were variable. Accordingly, our intent for this review was to pool these outcomes in order to reduce this bias and transparently report each case for appropriate assessment and application of our findings. In addition, Cusumano et al.’s [9] case series comprised 42 of the patients in our review and used a study end-point of death at 30 days, implicating that the true mortality rate of patients with COVID-19 and *S. aureus* co-infection may be higher if related complications necessitate an extended hospital course. Future high-quality clinical studies examining patient outcomes are warranted and of critical importance to further expand on the findings of our systematic review.

## Conclusion

In contrast to patients infected solely with COVID-19, co-infection with COVID-19 and *S. aureus* demonstrates a higher patient mortality rate during hospital admission. *S. aureus* co-infection in COVID-19 patients is predominantly healthcare-associated, and common hospital interventions for patients with severe COVID-19 infection may increase the risk for bacterial infection. Our findings emphasize the imperative of COVID-19 vaccination to prevent hospitalization for COVID-19 treatment and the subsequent susceptibility to hospital-acquired *S. aureus* co-infection.

## Supplementary Information

**Additional file 1: Table S1.** Search strategies, conducted between July 3, 2021, and July 16, 2021. Total results = 1922. **Table S2.** Joanna Briggs Quality Assessment for case reports included in the review. **Table S3:** Joanna Briggs Quality Assessment for case-series included in the review. **Table S4.** Joanna Briggs Quality Assessment for cohort studies included in the review. **Table S5.** Excluded articles after full-text analysis, with reason (n = 64).

## Data Availability

All data generated or analyzed during this study are included in this published article [and its Additional file 1].

## Abbreviations

COVID-19: Coronavirus disease 2019
SARS-CoV-2: Severe acute respiratory syndrome coronavirus 2
ARDS: Acute respiratory distress syndrome
*S. aureus*: *Staphylococcus aureus*
PRISMA: Preferred Reporting Items for Systematic Reviews and Meta-Analyses
SD: Standard deviation
MSSA: Methicillin-sensitive *Staphylococcus aureus*
MRSA: Methicillin-resistant *Staphylococcus aureus*
PVL-MSSA: Panton-Valentine Leukocidin methicillin-sensitive *Staphylococcus aureus*
NR: Not reported
Dx findings: Diagnostic findings
CXR: Chest x-ray
ICU: Intensive care unit
PCR: Polymerase chain reaction
CT: Computed tomography
LFTs: Liver function tests
ECMO: Extracorporeal membrane oxygenation
IFN: Interferon
MRI: Magnetic resonance imaging
