# Supplementary material for: Clinical outcomes in patients co-infected with COVID-19 and Staphylococcus aureus: a scoping review
Source: BMC Infect Dis. 2021 Sep 21;21:985. doi: 10.1186/s12879-021-06616-4 (PMC8453255; doi:10.1186/s12879-021-06616-4)
Supplement: Supplementary file 1 — Additional file 1: Table S1. Search strategies, conducted between July 3, 2021, and July 16, 2021. Total results = 1922. Table S2. Joanna Briggs Quality Assessment for case reports included in the review. Table S3: Joanna Briggs Quality Assessment for case-series included in the review. Table S4. Joanna Briggs Quality Assessment for cohort studies included in the review. Table S5. Excluded articles after full-text analysis, with reason (n = 64). [file 12879_2021_6616_MOESM1_ESM.docx]

**APPENDIX**

**Table S1:** Search strategies, conducted between July 3, 2021, and July 16, 2021. Total results = 1,922

| **PubMed**  (Total results = 100) | ((“Methicillin-Resistant Staphylococcus aureus”[Mesh] OR “Staphylococcus aureus”[Mesh] OR “Staphylococcal Infections”[Mesh] OR “methicillin-susceptible Staphylococcus aureus” [Title/Abstract] OR “vancomycin-intermediate Staphylococcus aureus” [Title/Abstract] OR “vancomycin-resistant Staphylococcus aureus” [Title/Abstract] OR “MRSA” [Title/Abstract] OR “MSSA” [Title/Abstract] OR “VISA” [Title/Abstract] OR “VRSA” [Title/Abstract])) AND ((“severe acute respiratory syndrome coronavirus 2” [Title/Abstract] OR “2019 Novel coronavirus” [Title/Abstract] OR “2019-nCoV infection” [Title/Abstract] OR “2019 novel coronavirus infection” [Title/Abstract] OR “coronavirus disease 2019 virus” [Title/Abstract] OR “wuhan coronavirus” [Title/Abstract] OR “coronavirus” [Title/Abstract] OR “Novel coronavirus” [Title/Abstract] OR “coronavirus disease” [Title/Abstract] OR “2019-ncov” [Title/Abstract] OR “COVID-19” [Mesh] OR “SARS-CoV-2” [Mesh])) |
| --- | --- |
| **Scopus**  (Total results = 644) | ((TITLE-ABS-KEY({“severe acute respiratory syndrome coronavirus 2”}) OR  TITLE-ABS-KEY ({“Novel coronavirus”}) OR TITLE-ABS-KEY ({“2019-nCoV infection”}) OR TITLE-ABS-KEY ({“2019 novel coronavirus infection”}) OR TITLE-ABS-KEY ({“coronavirus disease 2019 virus”}) OR TITLE-ABS-KEY ({“wuhan coronavirus”} ) OR TITLE-ABS-KEY ({“coronavirus”}) OR TITLE-ABS-KEY ({“Novel coronavirus”}) OR TITLE-ABS-KEY ({“coronavirus disease”}) OR TITLE-ABS-KEY ({“covid 2019”}) OR TITLE-ABS-KEY ({“covid-19”} ) OR TITLE-ABS-KEY ({“sars-cov2”})  OR  TITLE-ABS-KEY ({“2019-ncov”}))) AND ((TITLE-ABS-KEY ({“Methicillin-Resistant Staphylococcus aureus”}) OR TITLE-ABS-KEY ({“Staphylococcus aureus”}) OR TITLE-ABS-KEY ({“Staphylococcal Infections”}) OR TITLE-ABS-KEY ({“methicillin-susceptible Staphylococcus aureus”}) OR TITLE-ABS-KEY ({“MRSA”}) OR TITLE-ABS-KEY ({“vancomycin-resistant Staphylococcus aureus”}) OR TITLE-ABS-KEY ({“vancomycin-intermediate Staphylococcus aureus”}))) |
| **Ovid MEDLINE**  (Total results = 144) | (Methicillin-Resistant Staphylococcus aureus OR Staphylococcus aureus OR Staphylococcal Infections OR methicillin-susceptible Staphylococcus aureus OR MRSA OR VISA OR VRSA) AND (severe acute respiratory syndrome coronavirus 2 OR 2019 Novel coronavirus OR 2019-nCoV infection OR 2019 novel coronavirus infection OR coronavirus disease 2019 virus OR Novel coronavirus OR coronavirus disease OR covid-19 OR sars-cov2) |
| **CINAHL**  (Total results = 38) | (“Methicillin-Resistant Staphylococcus aureus” OR “Staphylococcus aureus” OR “Staphylococcal Infections” OR “methicillin-susceptible Staphylococcus aureus” OR “MRSA” OR “vancomycin-intermediate Staphylococcus aureus” OR “vancomycin-resistant Staphylococcus aureus”) AND (“severe acute respiratory syndrome coronavirus 2” OR “2019 Novel coronavirus” OR “2019-nCoV infection” OR “2019 novel coronavirus infection” OR “coronavirus disease 2019 virus” OR “Novel coronavirus” OR “coronavirus disease” OR “covid-19” OR “sars-cov2”) |
| **ScienceDirect**  (Total results = 168) | “severe acute respiratory syndrome coronavirus 2” OR “2019 Novel coronavirus” OR “2019-nCoV infection” OR “2019 novel coronavirus infection” OR “coronavirus disease 2019 virus” OR “Novel coronavirus” OR “coronavirus disease” OR “covid-19” OR “sars-cov2” AND Abs/Keywords: “Methicillin-Resistant Staphylococcus aureus” OR “Staphylococcus aureus” OR “Staphylococcal Infections” OR “methicillin-susceptible Staphylococcus aureus” OR “MRSA” OR “vancomycin-intermediate Staphylococcus aureus” OR “vancomycin-resistant Staphylococcus aureus” |
| **WHO Coronavirus Database**  (Total results = 265) | (tw:(“Methicillin-Resistant Staphylococcus aureus”)) OR (tw:(“Staphylococcus aureus”)) OR (tw:(“Staphylococcal Infections”)) OR (tw:(“methicillin-susceptible Staphylococcus aureus”)) OR (tw:(“MRSA”)) OR (tw:(“staph infections”)) OR (tw:(“vancomycin-resistant Staphylococcus aureus”)) OR (tw:(“vancomycin-intermediate Staphylococcus aureus”)) AND (tw:(“severe acute respiratory syndrome coronavirus 2”)) OR (tw:(“2019 Novel coronavirus”)) OR (tw:(“2019-nCoV infection”)) OR (tw:(“2019 novel coronavirus infection”)) OR (tw:(“coronavirus disease 2019 virus”)) OR (tw:(“Novel coronavirus”)) OR (tw:(“coronavirus disease”)) OR (tw:(“covid-19”)) OR (tw:(“sars-cov2”)) |
| **MedRxiv (preprint)**  (Multiple searches conducted; total results = 563) | "Staphylococcus aureus AND covid-19" [abstract/title] = 219 results  "Staphylococcus aureus AND sarscov-2" [abstract/title] = 6 results  "Staphylococcus aureus AND coronavirus" [abstract/title] = 209 results  "Staphylococcal AND sarscov-2" [abstract/title] = 0 results  “Staphylococcal AND covid-19” [abstract/title] = 29 results  "Staphylococcal AND coronavirus" [abstract/title] = 27 results  "MRSA AND covid-19" [abstract/title] = 70 results  "MRSA AND sarscov-2" [abstract/title] = 3 results  “MRSA AND coronavirus” [abstract/title] = 64 results |

**Table S2:** Joanna Briggs Quality Assessment for case reports included in the review

| First Author (Ref) | 1 | 2 | 3 | 4 | 5 | 6 | 7 | 8 | Total score |
| --- | --- | --- | --- | --- | --- | --- | --- | --- | --- |
| **Adachi** [1] | Yes | Yes | Yes | Yes | Yes | Yes | Yes | Yes | 8/8 |
| **Bagnato** [2] | Yes | Yes | Yes | Yes | Yes | Yes | Yes | Yes | 8/8 |
| **Chandran** [3] | No | Yes | Yes | Yes | Yes | Yes | Yes | Yes | 7/8 |
| **Chen** [4] | No | Yes | Yes | Yes | Yes | No | Yes | Yes | 6/8 |
| **Choudhury** [5] | Yes | Yes | Yes | Yes | Yes | No | Yes | Yes | 7/8 |
| **Duployez** [6] | Yes | Yes | Yes | Yes | Yes | Yes | Yes | Yes | 8/8 |
| **Edrada** [7] | Yes | Yes | Yes | Yes | No | Yes | No | Yes | 6/8 |
| **ElSeirafi** [8] | Yes | Yes | Yes | No | Yes | No | Yes | Yes | 6/8 |
| **Filocamo** [9] | Yes | Yes | Yes | Yes | Yes | Yes | Yes | Yes | 8/8 |
| **Hamzavi** [10] | Yes | No | Yes | Yes | Yes | No | Yes | Yes | 6/8 |
| **Hussain** [11] | Yes | Yes | Yes | Yes | Yes | Yes | Yes | Yes | 8/8 |
| **Levesque** [12] | Yes | No | Yes | Yes | Yes | No | Yes | Yes | 6/8 |
| **Mirza** [13] | Yes | Yes | Yes | Yes | Yes | No | No | Yes | 6/8 |
| **Patek** [14] | Yes | Yes | Yes | Yes | Yes | Yes | No | Yes | 7/8 |
| **Posteraro** [15] | Yes | Yes | Yes | Yes | Yes | Yes | Yes | Yes | 8/8 |
| **Rajdev** [16] | No | Yes | Yes | Yes | No | Yes | Yes | Yes | 6/8 |
| **Rajdev** [17] | Yes | Yes | Yes | Yes | Yes | Yes | Yes | Yes | 8/8 |
| **Randall** [18] | Yes | Yes | Yes | No | Yes | Yes | Yes | Yes | 7/8 |
| **Regazzoni** [19] | No | No | No | Yes | No | No | No | Yes | 2/8 |
| **Spannella** [20] | Yes | Yes | Yes | Yes | Yes | Yes | Yes | Yes | 8/8 |
| **Spoto** [21] | Yes | No | Yes | Yes | Yes | Yes | No | Yes | 6/8 |
| **Valga** [22] | Yes | Yes | No | Yes | Yes | No | Yes | Yes | 6/8 |

1. Were the patient’s demographic characteristics clearly described?
2. Was the patient’s history clearly described and presented as a timeline?
3. Was the current clinical condition of the patient on presentation clearly described?
4. Were diagnostic tests or assessment methods and the results clearly described?
5. Was the intervention(s) or treatment procedure(s) clearly described?
6. Was the post-intervention clinical condition clearly described?
7. Were adverse events (harms) or unanticipated events identified and described?
8. Does the case report provide takeaway lessons?

**Table S3:** Joanna Briggs Quality Assessment for case-series included in the review

| First Author (Ref) | 1 | 2 | 3 | 4 | 5 | 6 | 7 | 8 | 9 | 10 | Total Score |
| --- | --- | --- | --- | --- | --- | --- | --- | --- | --- | --- | --- |
| **Cusumano** [23] | Yes | Yes | Yes | No | Yes | Yes | Yes | Yes | Yes | Yes | 9/10 |
| **Hoshiyama** [24] | Yes | Yes | Yes | No | Yes | No | No | Yes | No | Yes | 6/10 |

1. Were there clear criteria for inclusion in the case series?
2. Was the condition measured in a standard, reliable way for all participants included in the case series?
3. Were valid methods used for identification of the condition for all participants included in the case series?
4. Did the case series have consecutive inclusion of participants?
5. Did the case series have complete inclusion of participants?
6. Was there clear reporting of the demographics of the participants in the study?
7. Was there clear reporting of clinical information of the participants?
8. Were the outcomes or follow up results of cases clearly reported?
9. Was there clear reporting of the presenting site(s)/clinic(s) demographic information?
10. Was statistical analysis appropriate?

**Table S4.** Joanna Briggs Quality Assessment for cohort studies included in the review

| First Author (Ref) | 1 | 2 | 3 | 4 | 5 | 6 | 7 | 8 | 9 | 10 | 11 | Total score |  |
| --- | --- | --- | --- | --- | --- | --- | --- | --- | --- | --- | --- | --- | --- |
| **De Pascale** [25] | Yes | Yes | Yes | Yes | Yes | No | Yes | Yes | No | No | Yes | 8/11 |  |
| **Ramos-Martinez** [26] | Yes | No | Yes | Yes | No | No | Yes | Yes | No | No | Yes | 6/11 |  |
| **Sharifipour** [27] | Yes | No | No | No | No | Yes | Yes | Yes | Yes | Yes | Yes | 7/11 |  |
| **Son** [28] | Yes | Yes | No | Yes | No | No | Yes | Yes | Yes | Yes | Yes | 8/11 |  |

1. Were the two groups similar and recruited from the same population?
2. Were the exposures measured similarly to assign people to both exposed and unexposed groups?
3. Was the exposure measured in a valid and reliable way?
4. Were confounding factors identified?
5. Were strategies to deal with confounding factors stated?
6. Were the groups/participants free of the outcome at the start of the study (or at the moment of exposure)?
7. Were the outcomes measured in a valid and reliable way?
8. Was the follow up time reported and sufficient to be long enough for outcomes to occur?
9. Was follow up complete, and if not, were the reasons to loss to follow up described and explored?
10. Were strategies to address incomplete follow up utilized?
11. Was appropriate statistical analysis used?

**Table S5.** Excluded articles after full-text analysis, with reason (n = 64)

| **Reason for exclusion** | **Articles (First author)** |
| --- | --- |
| Incidence/Prevalence study with no patient-specific outcome data (n = 57) | Acker [29], Adeiza [30], Aleman [31], Allou [32], Antinori [33], Bardi [34], Baskaran [35], Baunoch [36], Beovic [37], Bidulka [38], Calderaro [39], Coenen [40], Cheng [41], Contou [42], d’Humières [43], Denny [44], Dhesi [45], Elbaddi [46], Engsbro [47], Fattorini [48], Garcia-Vidal [49], Gerver [50], Giacobbe [51], Giacobbe [52], Grasselli [53], He [54], Intra [55], Karaba [56], Kewan [57], Kolenda [58], Kreitmann [59], Lardaro [60], Malberti [61], Manohar [62], May [63], Nasir [64], Nebreda-Mayoral [65], Nomani [66], Nori [67], Ouldali [68], Pickens [69], Posteraro [70], Punjabi [71], Qasim [72], Ramadan [73], Rothe [74], Ruiz-Bastian [75], Russell [76], Senok [77], Sepulveda [78], Silva [79], Singh [80], Tang [81], Verroken [82], Yang [83], Zhu [84], Zhong [85] |
| Not available in English (n = 3) | Fernandez [86], Konietzka [87], Luo [88] |
| History of *S. aureus* infection but no current infection (n = 2) | Lian [89], Qasim [72] |
| Genome analysis with no patient data (n = 2) | Marquez [90], Marquez [91] |

**References**

1. Adachi T, Chong J-M, Nakajima N, et al. Clinicopathologic and Immunohistochemical Findings from Autopsy of Patient with COVID-19, Japan. Emerging Infect Dis. 2020;26(9). doi:10.3201/eid2609.201353

2. Bagnato S, Boccagni C, Marino G, Prestandrea C, D’Agostino T, Rubino F. Critical illness myopathy after COVID-19. Int J Infect Dis. 2020;99:276-278. doi:10.1016/j.ijid.2020.07.072

3. Chandran S, Avari M, Cherian BP, Suarez C. COVID-19-associated Staphylococcus aureus cavitating pneumonia. BMJ Case Rep. 2021;14(6). doi:10.1136/bcr-2021-243726

4. Chen J, Tian C, Cheng X, Wang R, Zhou H, Zeng X. A case of asymptomatic SARS-CoV-2 infection followed by secondary community acquired pneumonia. Quant Imaging Med Surg. 2020;10(11):2208-2211. doi:10.21037/qims-20-847

5. Choudhury I, Han H, Manthani K, Gandhi S, Dabhi R. COVID-19 as a Possible Cause of Functional Exhaustion of CD4 and CD8 T-cells and Persistent Cause of Methicillin-Sensitive Staphylococcus aureus Bacteremia. Cureus. 2020;12(7):e9000. doi:10.7759/cureus.9000

6. Duployez C, Le Guern R, Tinez C, et al. Panton-Valentine Leukocidin-Secreting Staphylococcus aureus Pneumonia Complicating COVID-19. Emerging Infect Dis. 2020;26(8):1939-1941. doi:10.3201/eid2608.201413

7. Edrada EM, Lopez EB, Villarama JB, et al. First COVID-19 infections in the Philippines: a case report. Trop Med Health. 2020;48:21. doi:10.1186/s41182-020-00203-0

8. ElSeirafi MM, Hasan HM, Sridharan K, Zamoori A, Alkhawaja S, Pasha SAA. Efficacy and safety of tocilizumab in critically ill adults with COVID-19 infection in Bahrain: A report of 5 cases. Respir Med Case Rep. 2020;30:101139. doi:10.1016/j.rmcr.2020.101139

9. Filocamo G, Mangioni D, Tagliabue P, et al. Use of anakinra in severe COVID-19: A case report. Int J Infect Dis. 2020;96:607-609. doi:10.1016/j.ijid.2020.05.026

10. Hamzavi SS, Gholami MA, Sanaei Dashti A. A case of COVID 19 and staphylococcus coinfection. Arch Iran Med. 2020;23(8):568-569. doi:10.34172/aim.2020.62

11. Hussain A, Roberts N, Oo A. Prosthetic aortic valve endocarditis complicated by COVID-19 and hemorrhage. J Card Surg. 2020;35(6):1348-1350. doi:10.1111/jocs.14643

12. Lévesque V, Millaire É, Corsilli D, Rioux-Massé B, Carrier F-M. Severe immune thrombocytopenic purpura in critical COVID-19. Int J Hematol. 2020;112(5):746-750. doi:10.1007/s12185-020-02931-9

13. Mirza AA, Rad EJ, Mohabir PK. Cystic fibrosis and COVID-19: Care considerations. Respir Med Case Rep. 2020;31:101226. doi:10.1016/j.rmcr.2020.101226

14. Patek P, Corcoran J, Adams L, Khandhar P. SARS-CoV-2 Infection in a 2-Week-Old Male With Neutropenia. Clin Pediatr (Phila). 2020;59(9-10):918-920. doi:10.1177/0009922820920014

15. Posteraro B, Torelli R, Vella A, et al. Pan-Echinocandin-Resistant Candida glabrata Bloodstream Infection Complicating COVID-19: A Fatal Case Report. J Fungi (Basel). 2020;6(3). doi:10.3390/jof6030163

16. Rajdev K, Farr LA, Saeed MA, Hooten R, Baus J, Boer B. A Case of Extracorporeal Membrane Oxygenation as a Salvage Therapy for COVID-19-Associated Severe Acute Respiratory Distress Syndrome: Mounting Evidence. J Investig Med High Impact Case Rep. 2020;8:2324709620957778. doi:10.1177/2324709620957778

17. Rajdev K, Victor N, Buckholtz ES, et al. A Case of Guillain-Barré Syndrome Associated With COVID-19. J Investig Med High Impact Case Rep. 2020;8:2324709620961198. doi:10.1177/2324709620961198

18. Randall M, Minahan T, Mesisca M, Gnass S. Nosocomial methicillin-resistant Staphylococcus aureus bacteremia in incarcerated patients with severe COVID-19 infection. Am J Infect Control. 2020;48(12):1568-1569. doi:10.1016/j.ajic.2020.09.005

19. Regazzoni V, Loffi M, Garini A, Danzi GB. Glucocorticoid-Induced Bacterial Endocarditis in COVID-19 Pneumonia　- Something to Be Concerned About? Circ J. 2020;84(10):1887. doi:10.1253/circj.CJ-20-0462

20. Spannella F, Ristori L, Giulietti F, et al. A 95-year-old patient with unexpected coronavirus disease 2019 masked by aspiration pneumonia: a case report. J Med Case Reports. 2020;14(1):82. doi:10.1186/s13256-020-02432-7

21. Spoto S, Valeriani E, Riva E, et al. A Staphylococcus aureus Coinfection on a COVID-19 Pneumonia in a Breast Cancer Patient. Int J Gen Med. 2020;13:729-733. doi:10.2147/IJGM.S261760

22. Valga F, Vega-Diaz N, Monzon T, et al. Role of extended hemodialysis in COVID-19: a case report. Nefrología (English Edition). 2020;40(4):487-489. doi:10.1016/j.nefroe.2020.05.002

23. Cusumano JA, Dupper AC, Malik Y, et al. Staphylococcus aureus Bacteremia in Patients Infected With COVID-19: A Case Series. Open Forum Infect Dis. 2020;7(11):ofaa518. doi:10.1093/ofid/ofaa518

24. Hoshiyama T, Wada T, Nihonyanagi S, et al. Clinical and Microbiological Features of Asymptomatic SARS-CoV-2 Infection and Mild COVID-19 in Seven Crewmembers of a Cruise Ship. Intern Med. 2020;59(24):3135-3140. doi:10.2169/internalmedicine.5601-20

25. De Pascale G, De Maio F, Carelli S, et al. Staphylococcus aureus ventilator-associated pneumonia in patients with COVID-19: clinical features and potential inference with lung dysbiosis. Crit Care. 2021;25(1):197. doi:10.1186/s13054-021-03623-4

26. Ramos-Martínez A, Fernández-Cruz A, Domínguez F, et al. Hospital-acquired infective endocarditis during Covid-19 pandemic. Infection Prevention in Practice. 2020;2(3):100080. doi:10.1016/j.infpip.2020.100080

27. Sharifipour E, Shams S, Esmkhani M, et al. Evaluation of bacterial co-infections of the respiratory tract in COVID-19 patients admitted to ICU. BMC Infect Dis. 2020;20(1):646. doi:10.1186/s12879-020-05374-z

28. Son H-J, Kim T, Lee E, et al. Risk factors for isolation of multi-drug resistant organisms in coronavirus disease 2019 pneumonia: a multicenter study. Am J Infect Control. June 2021. doi:10.1016/j.ajic.2021.06.005

29. Acker KP, Schertz K, Abramson EL, DeLaMora P, Salvatore CM, Han J-Y. Infectious diseases diagnoses of children admitted with symptoms of coronavirus disease 2019 during an outbreak in new york city. Clin Pediatr (Phila). 2020;59(14):1293-1295. doi:10.1177/0009922820944399

30. Adeiza SS, Shuaibu AB, Shuaibu GM. Random effects meta-analysis of COVID-19/S. aureus partnership in co-infection. GMS Hyg Infect Control. 2020;15:Doc29. doi:10.3205/dgkh000364

31. Aleman VD, Garnett Carbajal JA, Alvarez Velazquez FA, et al. Incidence of coinfection in patients with covid-19. Chest. 2020;158(4):A343. doi:10.1016/j.chest.2020.08.340

32. Allou N, Larsen K, Dubernet A, et al. Co-infection in patients with hypoxemic pneumonia due to COVID-19 in Reunion Island. Medicine. 2021;100(4):e24524. doi:10.1097/MD.0000000000024524

33. Antinori S, Galimberti L, Milazzo L, Ridolfo AL. Bacterial and fungal infections among patients with SARS-CoV-2 pneumonia. Infez Med. 2020;28(suppl 1):29-36.

34. Bardi T, Pintado V, Gomez-Rojo M, et al. Nosocomial infections associated to COVID-19 in the intensive care unit: clinical characteristics and outcome. Eur J Clin Microbiol Infect Dis. 2021;40(3):495-502. doi:10.1007/s10096-020-04142-w

35. Baskaran V, Lawrence H, Lansbury L, et al. Co-infection in critically ill patients with COVID-19: An observational cohort study from England. medRxiv. October 2020. doi:10.1101/2020.10.27.20219097

36. Baunoch D, Wolfe A, Wang D, et al. Co-occurrence of SARS-CoV-2 and Respiratory Pathogens in the Frail Elderly. medRxiv. June 2020. doi:10.1101/2020.06.24.20138941

37. Beović B, Doušak M, Ferreira-Coimbra J, et al. Antibiotic use in patients with COVID-19: a “snapshot” Infectious Diseases International Research Initiative (ID-IRI) survey. J Antimicrob Chemother. 2020;75(11):3386-3390. doi:10.1093/jac/dkaa326

38. Bidulka P, Iwagami M, Mansfield KE, et al. Comparisons of Staphylococcus aureus infection and other outcomes between users of angiotensin-converting-enzyme inhibitors and angiotensin II receptor blockers: lessons for COVID-19 from a nationwide cohort study. Wellcome Open Res. 2020;5:77. doi:10.12688/wellcomeopenres.15873.1

39. Calderaro A, Buttrini M, Montecchini S, et al. Detection of SARS-CoV-2 and Other Infectious Agents in Lower Respiratory Tract Samples Belonging to Patients Admitted to Intensive Care Units of a Tertiary-Care Hospital, Located in an Epidemic Area, during the Italian Lockdown. Microorganisms. 2021;9(1). doi:10.3390/microorganisms9010185

40. Coenen S, de la Court JR, Buis DTP, et al. Low frequency of community-acquired bacterial co-infection in patients hospitalized for COVID-19 based on clinical, radiological and microbiological criteria; a retrospective cohort study. medRxiv. July 2021. doi:10.1101/2021.06.23.21259020

41. Cheng LS-K, Chau SK-Y, Tso EY-K, et al. Bacterial co-infections and antibiotic prescribing practice in adults with COVID-19: experience from a single hospital cluster. Ther Adv Infect Dis. 2020;7:2049936120978095. doi:10.1177/2049936120978095

42. Contou D, Claudinon A, Pajot O, et al. Bacterial and viral co-infections in patients with severe SARS-CoV-2 pneumonia admitted to a French ICU. Ann Intensive Care. 2020;10(1):119. doi:10.1186/s13613-020-00736-x

43. d’Humières C, Patrier J, Lortat-Jacob B, et al. Two original observations concerning bacterial infections in COVID-19 patients hospitalized in intensive care units during the first wave of the epidemic in France. PLoS One. 2021;16(4):e0250728. doi:10.1371/journal.pone.0250728

44. Denny S, Rawson TM, Hart P, et al. Bacteraemia variation during the COVID-19 pandemic; a multi-centre UK secondary care ecological analysis. BMC Infect Dis. 2021;21(1):556. doi:10.1186/s12879-021-06159-8

45. Dhesi Z, Enne VI, Brealey D, et al. Organisms causing secondary pneumonias in COVID-19 patients at 5 UK ICUs as detected with the FilmArray test. medRxiv. June 2020. doi:10.1101/2020.06.22.20131573

46. Elabbadi A, Turpin M, Gerotziafas GT, Teulier M, Voiriot G, Fartoukh M. Bacterial coinfection in critically ill COVID-19 patients with severe pneumonia. Infection. 2021;49(3):559-562. doi:10.1007/s15010-020-01553-x

47. Engsbro AL, Israelsen SB, Pedersen M, et al. Predominance of hospital-acquired bloodstream infection in patients with Covid-19 pneumonia. Infect Dis (Lond). 2020;52(12):919-922. doi:10.1080/23744235.2020.1802062

48. Fattorini L, Creti R, Palma C, Pantosti A, Unit of Antibiotic Resistance and Special Pathogens, Unit of Antibiotic Resistance and Special Pathogens of the Department of Infectious Diseases, Istituto Superiore di Sanità, Rome. Bacterial coinfections in COVID-19: an underestimated adversary. Ann Ist Super Sanita. 2020;56(3):359-364. doi:10.4415/ANN_20_03_14

49. Garcia-Vidal C, Sanjuan G, Moreno-García E, et al. Incidence of co-infections and superinfections in hospitalized patients with COVID-19: a retrospective cohort study. Clin Microbiol Infect. 2021;27(1):83-88. doi:10.1016/j.cmi.2020.07.041

50. Gerver SM, Guy R, Wilson K, et al. National surveillance of bacterial and fungal co- and secondary infection in COVID-19 patients in England – Lessons from the first wave. Clin Microbiol Infect. June 2021. doi:10.1016/j.cmi.2021.05.040

51. Giacobbe DR, Battaglini D, Enrile EM, et al. Incidence and Prognosis of Ventilator-Associated Pneumonia in Critically Ill Patients with COVID-19: A Multicenter Study. J Clin Med. 2021;10(4). doi:10.3390/jcm10040555

52. Giacobbe DR, Battaglini D, Ball L, et al. Bloodstream infections in critically ill patients with COVID-19. Eur J Clin Invest. 2020;50(10):e13319. doi:10.1111/eci.13319

53. Grasselli G, Scaravilli V, Mangioni D, et al. Hospital-Acquired Infections in Critically Ill Patients With COVID-19. Chest. April 2021. doi:10.1016/j.chest.2021.04.002

54. He F, Xia X, Nie D, et al. Respiratory bacterial pathogen spectrum among COVID-19 infected and non-COVID-19 virus infected pneumonia patients. Diagn Microbiol Infect Dis. 2020;98(4):115199. doi:10.1016/j.diagmicrobio.2020.115199

55. Intra J, Sarto C, Beck E, Tiberti N, Leoni V, Brambilla P. Bacterial and fungal colonization of the respiratory tract in COVID-19 patients should not be neglected. Am J Infect Control. 2020;48(9):1130-1131. doi:10.1016/j.ajic.2020.06.185

56. Karaba SM, Jones G, Helsel T, et al. Prevalence of Co-infection at the Time of Hospital Admission in COVID-19 Patients, A Multicenter Study. Open Forum Infect Dis. 2021;8(1):ofaa578. doi:10.1093/ofid/ofaa578

57. Kewan T, Chawla S, Saleem T, Afzal S, Saand A, Alqaisi S. Superinfections in patients infected with covid-19: a single-center experience. Chest. 2020;158(4):A350. doi:10.1016/j.chest.2020.08.347

58. Kolenda C, Ranc A-G, Boisset S, et al. Assessment of Respiratory Bacterial Coinfections Among Severe Acute Respiratory Syndrome Coronavirus 2-Positive Patients Hospitalized in Intensive Care Units Using Conventional Culture and BioFire, FilmArray Pneumonia Panel Plus Assay. Open Forum Infect Dis. 2020;7(11):ofaa484. doi:10.1093/ofid/ofaa484

59. Kreitmann L, Monard C, Dauwalder O, Simon M, Argaud L. Early bacterial co-infection in ARDS related to COVID-19. Intensive Care Med. 2020;46(9):1787-1789. doi:10.1007/s00134-020-06165-5

60. Lardaro T, Wang AZ, Bucca A, et al. Characteristics of COVID-19 patients with bacterial coinfection admitted to the hospital from the emergency department in a large regional healthcare system. J Med Virol. 2021;93(5):2883-2889. doi:10.1002/jmv.26795

61. Malberti F, Pecchini P, Marchi G, Foramitti M. When a nephrology ward becomes a COVID-19 ward: the Cremona experience. J Nephrol. 2020;33(4):625-628. doi:10.1007/s40620-020-00743-y

62. Manohar P, Loh B, Nachimuthu R, Hua X, Welburn SC, Leptihn S. Secondary bacterial infections in patients with viral pneumonia. Front Med (Lausanne). 2020;7:420. doi:10.3389/fmed.2020.00420

63. May A, Swetenham N, Pandey M, Taylor V, Hughes H, Underwood J. P197 Bacterial and fungal respiratory co-infection among patients admitted to ICU with COVID-19: a retrospective cohort study in a UK hospital. In: Infection, Co-Infection and Chronic Infection. BMJ Publishing Group Ltd and British Thoracic Society; 2021:A196-A197. doi:10.1136/thorax-2020-BTSabstracts.342

64. Nasir N, Rehman F, Omair SF. Risk factors for bacterial infections in patients with moderate to severe COVID-19: A case control study. medRxiv. January 2021. doi:10.1101/2021.01.09.21249498

65. Nebreda-Mayoral T, Miguel-Gómez MA, March-Rosselló GA, et al. Bacterial/fungal infection in hospitalized patients with COVID-19 in a tertiary hospital in the Community of Castilla y León, Spain. Enfermedades infecciosas y microbiologia clinica (English ed). December 2020. doi:10.1016/j.eimc.2020.11.003

66. Nomani M, Varahram M, Tabarsi P, et al. Decreased neutrophil‐mediated bacterial killing in COVID‐19 patients. Scand J Immunol. June 2021. doi:10.1111/sji.13083

67. Nori P, Cowman K, Chen V, et al. Bacterial and fungal coinfections in COVID-19 patients hospitalized during the New York City pandemic surge. Infect Control Hosp Epidemiol. 2021;42(1):84-88. doi:10.1017/ice.2020.368

68. Ouldali N, Yang DD, Madhi F, et al. Factors Associated With Severe SARS-CoV-2 Infection. Pediatrics. 2021;147(3):e2020023432. doi:10.1542/peds.2020-023432

69. Pickens CO, Gao CA, Cuttica M, et al. Bacterial superinfection pneumonia in SARS-CoV-2 respiratory failure. medRxiv. January 2021. doi:10.1101/2021.01.12.20248588

70. Posteraro B, De Angelis G, Menchinelli G, et al. Risk Factors for Mortality in Adult COVID-19 Patients Who Develop Bloodstream Infections Mostly Caused by Antimicrobial-Resistant Organisms: Analysis at a Large Teaching Hospital in Italy. J Clin Med. 2021;10(8). doi:10.3390/jcm10081752

71. Punjabi CD, Madaline T, Gendlina I, Chen V, Nori P, Pirofski L-A. Prevalence of methicillin-resistant Staphylococcus aureus (MRSA) in respiratory cultures and diagnostic performance of the MRSA nasal polymerase chain reaction (PCR) in patients hospitalized with coronavirus disease 2019 (COVID-19) pneumonia. Infect Control Hosp Epidemiol. August 2020:1-2. doi:10.1017/ice.2020.440

72. Qasim A, Mansour M, Kousa O, et al. A case of coronavirus disease 2019 in acquired immunodeficiency syndrome patient: a case report and review of the literature. Intractable Rare Dis Res. 2020;9(4):256-259. doi:10.5582/irdr.2020.03081

73. Ramadan HK-A, Mahmoud MA, Aburahma MZ, et al. Predictors of Severity and Co-Infection Resistance Profile in COVID-19 Patients: First Report from Upper Egypt. Infect Drug Resist. 2020;13:3409-3422. doi:10.2147/IDR.S272605

74. Rothe K, Feihl S, Schneider J, et al. Rates of bacterial co-infections and antimicrobial use in COVID-19 patients: a retrospective cohort study in light of antibiotic stewardship. Eur J Clin Microbiol Infect Dis. 2021;40(4):859-869. doi:10.1007/s10096-020-04063-8

75. Ruiz-Bastián M, Falces-Romero I, Ramos-Ramos JC, de Pablos M, García-Rodríguez J. Bacterial co-infections in covid-19 pneumonia in a tertiary care hospital: surfing the first wave. Diagn Microbiol Infect Dis. July 2021:115477. doi:10.1016/j.diagmicrobio.2021.115477

76. Russell CD, Fairfield CJ, Drake TM, et al. Co-infections, secondary infections, and antimicrobial use in patients hospitalised with COVID-19 during the first pandemic wave from the ISARIC WHO CCP-UK study: a multicentre, prospective cohort study. Lancet Microbe. June 2021. doi:10.1016/S2666-5247(21)00090-2

77. Senok A, Alfaresi M, Khansaheb H, et al. Coinfections in Patients Hospitalized with COVID-19: A Descriptive Study from the United Arab Emirates. Infect Drug Resist. 2021;14:2289-2296. doi:10.2147/IDR.S314029

78. Sepulveda J, Westblade LF, Whittier S, et al. Bacteremia and Blood Culture Utilization during COVID-19 Surge in New York City. J Clin Microbiol. 2020;58(8). doi:10.1128/JCM.00875-20

79. Silva DL, Lima CM, Magalhães VCR, et al. Fungal and bacterial coinfections increase mortality of severely ill COVID-19 patients. J Hosp Infect. 2021;113:145-154. doi:10.1016/j.jhin.2021.04.001

80. Singh V, Upadhyay P, Reddy J, Granger J. SARS-CoV-2 respiratory co-infections: Incidence of viral and bacterial co-pathogens. Int J Infect Dis. 2021;105:617-620. doi:10.1016/j.ijid.2021.02.087

81. Tang H, Zhao Z, Zhang X, et al. Analysis of pathogens and risk factors of secondary pulmonary infection in patients with COVID-19. Microb Pathog. 2021;156:104903. doi:10.1016/j.micpath.2021.104903

82. Verroken A, Scohy A, Gérard L, Wittebole X, Collienne C, Laterre P-F. Co-infections in COVID-19 critically ill and antibiotic management: a prospective cohort analysis. Crit Care. 2020;24(1):410. doi:10.1186/s13054-020-03135-7

83. Yang S, Hua M, Liu X, et al. Bacterial and fungal co-infections among COVID-19 patients in intensive care unit. Microbes Infect. 2021;23(4-5):104806. doi:10.1016/j.micinf.2021.104806

84. Zhu X, Ge Y, Wu T, et al. Co-infection with respiratory pathogens among COVID-2019 cases. Virus Res. 2020;285:198005. doi:10.1016/j.virusres.2020.198005

85. Zhong H, Wang Y, Shi Z, et al. Characterization of Microbial Co-infections in the Respiratory Tract of hospitalized COVID-19 patients. medRxiv. July 2020. doi:10.1101/2020.07.02.20143032

86. Fernández P, Moreno L, Yagüe G, Andreu E, Jara R, Segovia M. Colonization by multidrug-resistant microorganisms in ICU patients during the COVID-19 pandemic. Med Intensiva. 2021;45(5):313-315. doi:10.1016/j.medine.2021.04.006

87. Konietzka C, Schneider-Kruse M, Knaack D, Krüger C, Layer F, Endmann M. [Toxic shock syndrome due to Staphylococcus aureus in a small child, a (clinical or laboratory chemical) visual diagnosis?]. Monatsschr Kinderheilkd. November 2020:1-5. doi:10.1007/s00112-020-01075-8

88. Luo X, Hou H, Zhang B, et al. Clinical characteristics of 95 COVID-19 patients with bacterial and fungal infections. Chinese Journal of Microbiology and Immunology (China). January 2021.

89. Lian J, Jin X, Hao S, et al. Epidemiological, clinical, and virological characteristics of 465 hospitalized cases of coronavirus disease 2019 (COVID-19) from Zhejiang province in China. Influenza Other Respi Viruses. 2020;14(5):564-574. doi:10.1111/irv.12758

90. Márquez S, Prado-Vivar B, Guadalupe JJ, et al. Metagenome of a Bronchoalveolar Lavage Fluid Sample from a Confirmed COVID-19 Case in Quito, Ecuador, Obtained Using Oxford Nanopore MinION Technology. Microbiol Resour Announc. 2020;9(41). doi:10.1128/MRA.00996-20

91. Marquez S, Prado-Vivar B, Guadalupe JJ, et al. Genome sequencing of the first SARS-CoV-2 reported from patients with COVID-19 in Ecuador. medRxiv. June 2020. doi:10.1101/2020.06.11.20128330
